# Successful Treatment of Pneumothorax in a Dog With Sterile Pleural Fibrosis Caused by Chylothorax

**DOI:** 10.3389/fvets.2019.00278

**Published:** 2019-08-22

**Authors:** Sina Rehbein, George Manchi, Achim D. Gruber, Barbara Kohn

**Affiliations:** ^1^Clinic for Small Animals, Freie Universität, Berlin, Germany; ^2^Department of Veterinary Medicine, Institute of Veterinary Pathology, Freie Universität, Berlin, Germany

**Keywords:** chylothorax, canine, pleural fibrosis, unexpandable lung, pneumothorax ex vacuo, NSAID, meloxicam

## Abstract

A 2-year-old, 12 kg, intact male crossbreed dog was presented with respiratory distress, exercise intolerance, and gagging. Plain thoracic radiographs revealed severe pleural effusion. Although bilateral needle thoracocentesis and chest tube placement were performed, no re-expansion of the lung lobes occurred. Pleural effusion was of chylous quality and led to lung entrapment. Computer tomography revealed a highly atrophic and atelectatic right middle lung lobe. The remaining lung lobes were only expanded to ~40%. Visceral pleura and pericardium showed a heterogeneous thickening consistent with pleural fibrosis. Partial pericardiectomy with resection of the middle lung lobe through a right lateral thoracotomy was performed. Ligation of the thoracic duct and ablation of the cisterna chyli was achieved through a single paracostal approach. Histopathology revealed chronic-active proliferative beginning granulomatous pleuritis, fibrotic pericarditis, and partial coagulative necrosis with incomplete granulomatous sequestration in the resected middle lung lobe. Chylothorax resolved after surgical intervention. Active pleural effusion resolved, and lung entrapment changed to trapped lung disease. The remaining lung lobes re-expanded to ~80% over the following 6 days. The dog was discharged 10 days later. Mild to moderate pleural effusion of non-chylic quality was present during the following 4 months. Meloxicam was administered for 4 months because of its anti-fibrotic and anti-inflammatory properties. Fifteen months later, thoracic radiographs revealed full radiologic expansion of the lungs with persistent mild pleural fibrosis. To the authors' knowledge, this is the first case report of pneumothorax due pleural fibrosis caused by chylothorax in a dog with an excellent clinical outcome.

## Background

Pleural fibrosis is a rarely described pathological condition in dogs and cats. To the authors' knowledge, only 5 canine and 22 feline cases have been reported (Table 1). Previously, this condition was termed constrictive pleuritis. Due to pleural inflammation, a progressive constriction of lung parenchyma occurs, called lung entrapment. This condition may result in severe respiratory distress (6). In general, pleural fibrosis results from inflammatory processes within the thoracic cavity combined with active pleural effusions. Chylothorax and hemothorax are described in dogs with pleural fibrosis (Table 1). Radiologically, pleural fibrosis should be suggested if the lungs appears rounded accompanied with pleural effusion or if pneumothorax occurs after thoracocentesis (9). Decortication is recommended in case of severe respiratory distress. Complications like pneumothorax, tracheal tears, re-expansion pulmonary edemas, or death may occur (5, 7, 10). Anti-inflammatory doses of prednisone were given in cats with severe pleural fibrosis. The results were variable (6). However, prognosis in dogs and cats with pleural fibrosis is poor. In the literature, mortality rate in dogs and cats amounts 70% (17/24 dogs and cats with known outcome; Table 1).

**Table 1:** Summary of current literature about fibrosing pleuritis in dogs and cats.

**References**	**Number of patients**	**Species**	**Signalment**	**Duration of clinical signs**	**Diagnosis**	**Treatment**	**Outcome**
Stevenson et al. (1)	One	Cat	2-year-old, male DSH cat	4 weeks	Feline infectious peritonitis	N.a.	Cat died
Chastain et al. (2)	One	Dog	5-year-old, male Labrador Retriever	Unclear	Granulomatous pleuritis, pericardial effusion: *Pasteurella multocida*; 8 months later pleural effusion: *Actinomyces bovis*	Pericardiocentesis, antibiotics; 8 months later: thoracocentesis; euthanasia	Euthanasia
Read (3)	One	Dog	4-year-old, male Labrador	1 day	Traumatic hemothorax, constrictive pleuritis, prednisone	Decortication	Successful outcome; dog 8 months later clinically unremarkable
Glennon et al. (4)	One	Cat	18-year-old, castrated male American short-hair	Unclear	Hyperthyroidism, chylothorax, constrictive pleuritis	Thoracotomy, decortication	Euthanasia 5 days after surgery
Fossum et al. (5)	Five	Dog	2-year-old, sexually intact mixed-breed dog	6 months	Chylothorax, pleural fibrosis, *Serratia* spp.	Antibiotic therapy on the basis of sensitivity testing	Dog died 3 days after admission
		Dog	3-year-old, sexually intact male Shetland Sheepdog	4 days	Chylothorax, fibrosing pleuritis	Surgical exploration euthanasia due to guarded prognosis	Euthanasia
		Cat	5-year-old, neutered male Himalayan cat	18 months	Chylothorax, fibrosing pleuritis	Antibiotics, needle thoracocentesis, surgical exploration euthanasia due to guarded prognosis	Euthanasia
		Cat	5-year-old, neutered male DSH cat	3 weeks	Chylothorax, fibrosing pleuritis	Euthanasia	Euthanasia
		Cat	4-year-old, spayed DSH cat	2 weeks	FeLV positive, lymphoblastic lymphosarcoma, fibrosing pleuritis	Chemotherapy, needle thoracocentesis, bilateral placement of perforated plastic sheetings in the diaphragm	Euthanasia 4.5 months after surgery
		Cat	3-year-old, spayed DSH cat	8 months chylothorax, 3 weeks dyspnea and lethargy	Chylothorax, fibrosing pleuritis	Needle thoracocentesis, surgical exploration, bilateral placement of perforated plastic sheetings	Cat died 72 h after surgery
		Cat	5-year-old, neutered male DSH cat	9 months	Chylothorax, fibrosing pleuritis	Surgical exploration, decortication, partial lobectomy, chest tube placement, pulmonary edema	Cat died 4 h after surgery
Suess et al. (6)	Ten [one cat published in Glennon et al. (4)]	Cat	12-year-old, male castrated DSH cat	1 week	Chylothorax, HCM, fibrosing pleuritis	Captopril, furosemide, acetylsalicylic acid	Euthanasia 12 weeks after initial presentation
		Cat	8-year-old, female spayed DSH cat	13 weeks	Chylothorax, fibrosing pleuritis	Periodic thoracocentesis, low-fat diet, exercise restriction	Euthanasia 52 weeks after initial presentation
		Cat	7-year-old, male castrated Himalayan	4 weeks	Chylothorax, fibrosing pleuritis	Thoracic duct ligation	Resolution of chylothorax; non-chylous effusion; cat died 16 weeks after initial presentation
		Cat	4-year-old, male castrated DSH cat	3 weeks	Chylothorax, fibrosing pleuritis	Thoracic duct ligation, prednisone	Resolution of chylothorax; alive 82 weeks after initial presentation
		Cat	3-year-old, male castrated Himalayan	30 weeks	Chylothorax, fibrosing pleuritis	Prednisone	Resolution of chylothorax; alive 69 weeks after initial presentation
		Cat	5-year-old, female spayed DSH	17 weeks	Chylothorax, fibrosing pleuritis	Periodic thoracocentesis, low-fat diet, exercise restriction	Euthanasia 2 days after initial presentation
		Cat	7-year-old, male castrated DLH cat	5 weeks	Chylothorax, fibrosing pleuritis	Thoracic duct ligation, prednisone	Resolution of chylothorax; non-chylous effusion; euthanasia 8 weeks after initial presentation
		Cat	4-year-old, male castrated DSH	9 weeks	Chylothorax, fibrosing pleuritis	Thoracic duct ligation, prednisone	Resolution of chylothorax: non-chylous effusion; euthanasia 30 weeks after initial presentation
		Cat	12-year-old, female spayed DSH cat	1 day	Chylothorax, fibrosing pleuritis	Thoracic duct ligation, prednisone, passive peritoneal lavage	Resolution of chylothorax; non-chylous effusion; euthanasia 3 weeks after initial presentation
Fossum et al. (7)	Three	Cat	N.a.	2 weeks to 1 year	Chylothorax, severe fibrosing pleuritis	Thoracotomy with thoracic duct ligation, pericardiectomy, two cats received decortication, complications: two cats had pneumothorax, one cat had tracheal tear	Pneumothorax resolved in both cats with decortication; remaining cats unclear
	Two	Cat	N.a.	2 weeks to 1 year	Chylothorax, moderate fibrosing pleuritis		
	One	Dog	N.a.	2 weeks to 1 year	Chylothorax, mild fibrosing pleuritis	Thoracotomy with thoracic duct ligation, pericardiectomy	Chylothorax resolved
Lafond et al. (8)	One	Cat	6-year-old, spayed female Himalayan cat	2 months	Chylothorax, constrictive pleuritis	Omentalization of the thorax, partial decortication; complication: transient pneumothorax	Full recovery; clinically normal 13 months after surgery

*N.a., not approved; DSH, domestic shorthair; DLH, domestic longhair*.

The aim of this case report is to describe clinical presentation, imaging alterations, treatment and outcome in a dog with pneumothorax caused by pleural fibrosis after chylothorax.

## Case Presentation

A 2-year-old, weighing 12 kg, intact male crossbreed dog was referred to the Small Animal Clinic at Freie Universität Berlin with respiratory distress over the past 5 days. The owners observed a reduced appetite, exercise intolerance and gagging 2 weeks prior to admission. Pre-treatment with amoxicillin/clavulanic acid (20 mg/kg q12h PO) for 3 days was initiated by the referring veterinarian, but no clinical improvement occurred.

On presentation, the dog showed moderate tachypnea with a resting respiratory rate of 60 breaths per minute. Thoracic auscultation revealed muffled heart and significantly reduced lung sounds in the ventral thorax. On both thoracic sides, percussion produced a dampened, hyporesonant sound. All other clinical parameters were unremarkable. Hematology and blood chemistry were unremarkable with the exception of mild leukocytosis (19.2^*^10^9^/L, reference interval [RI] 5.6–14.0^*^10^9^/L) and thrombocytosis (456^*^10^9^/L, RI 165–400^*^10^9^/L).

**Figure 1:**
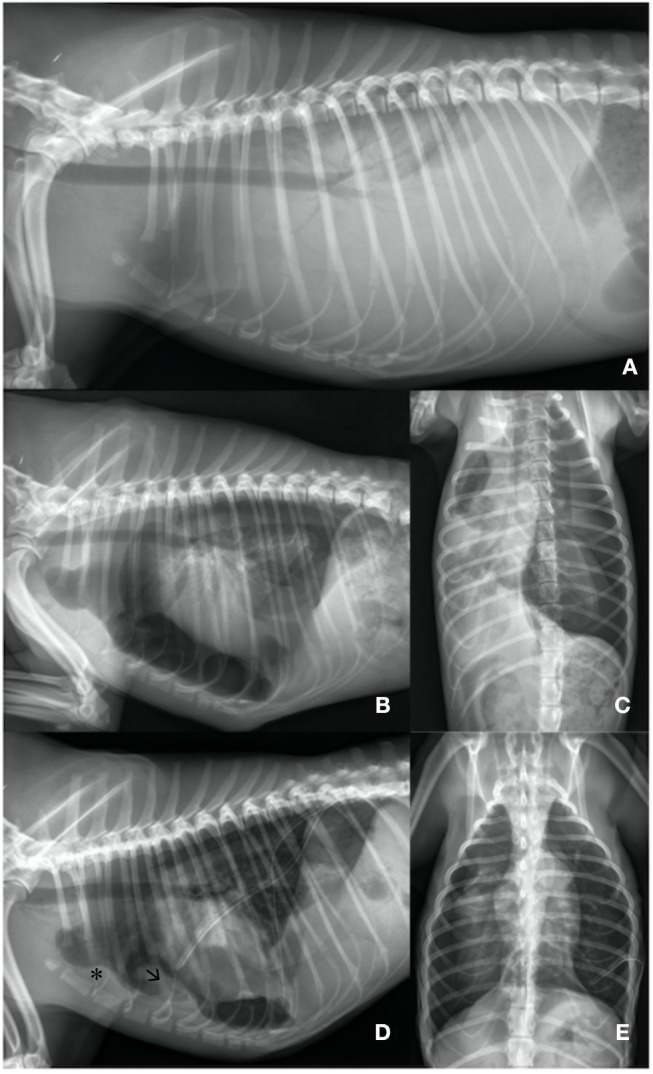
**(A)** Day 1: Lateral left sided thoracic radiograph view: Markedly increased soft- tissue opacity with completely silhouetted cardiac silhouette and retracted lung lobes due to a severe pleural effusion. **(B,C)** Day 1: Lateral left sided and ventrodorsal thoracic radiograph view: After needle thoracocentesis lung lobes were severely rounded and expanded to ~40%. Heterogenous pleural thickening is present. The heart is detached from the sternum. An iatrogenic pneumothorax was suspected. **(D,E)** Day 3: Lateral left sided and ventrodorsal thoracic radiograph view: Chest tubes are placed bilaterally. Lung lobes are re-expanded to 80% on the left and to 60% on the right side. Sternal lymphnode (asterisk) is prominent and a precardial mass-effect (arrow) is seen. The latter is suggestive for fibrotic tissue.

Stabilization was initialized with oxygen supplementation by mask with a flow rate of 2 L/min. Pulse oximetry revealed a SpO_2_ of 80%. The dog was sedated with butorphanol (Butorgesic, cp-pharma®, 0.3 mg/kg IV) and midazolam (Midazolam-ratiopharm®, 15 mg/3 ml, 0.2 mg/kg IV). After initial stabilization, a left sided thoracic radiograph revealed severe pleural effusion (Figure 1A).

750 ml on the left and 600 ml on the right thoracic side of a white-milky fluid was aspirated by needle thoracocentesis with a closed device under ultrasound guidance. Suction was performed until a negative pressure was obtained. A balanced crystalloid infusion (Sterofundin, B. Braun Melsungen AG; 2 ml/kg/h IV) and further oxygen supplementation were given. Clinical status improved. Breathing changed to a costoabdominal pattern with 32 breaths per minute and pulse oximetry revealed a SpO_2_ of 90%. A second thoracic radiograph was made. All lung lobes appeared rounded with severely reduced volume and were collapsed and retracted from the parietal pleura. Marked pleural thickening of all lung lobes was present (Figures 1B,C). Pleural fibrosis was suggested (9).

Because the lung lobes did not re-expand after thoracocentesis, iatrogenic pneumothorax was suspected. Therefore, a second needle thoracocentesis on the left side was performed. Negative pressure was still present. As a next step, chest tubes with non-return valves (Heimlich type, LEO-ventil, Eickemeyer) were placed bilaterally under general anesthesia (induction with propofol (Narcofol, 10 mg/ml, cp-pharma®); maintenance with isoflurane (Isofluran cp, cp-pharma®); constant rate infusion with fentanyl (Fentadon®, 50 μg/ml, Dechra, 0.01 mg/kg/h). During this intervention intermittent positive pressure ventilation was performed. Chest tubes were checked for possible leakage by active suction q24h and thoracic radiographs.

Effusion was diagnosed as exudative chylothorax [cholesterol effusion: 0.89 mmol/L, cholesterol serum content 3.43 mmol/L; triglycerides effusion: 6.51 mmol/L, triglycerides serum content 0.53 mmol/L; cholesterol: triglyceride ratio 0.14 (<1)]. Aerobic and anaerobic bacterial cultures were negative. Differential cell count showed a neutrophilia (16.3^*^10^9^/L; RI 3.6–12.0^*^10^9^/L), a monocytosis (2.3^*^10^9^/L, RI 0.0–1.5^*^10^9^/L), and a lymphopenia (0.38^*^10^9^/L, RI 0.7–4.8^*^10^9^/L). Laboratory results were consistent with the presence of chylothorax and chronic inflammation. Abdominal radiographs and echocardiography were unremarkable. Despite clinical improvement at day three, there was no re-expansion of the lung lobes (Figures 1D,E). Computer tomography was performed under general anesthesia (premedication: 0.2 mg/kg midazolam (Midazolam-ratiopharm®, 15 mg/3 ml), combination product of 0.25 mg/kg levomethadonhydrochloride and 0.013 mg/kg fenpipramidhydhrochloride (L-Polamivet® 2.5/0.125 mg/ml, MSD); induction: 4 mg/kg propofol IV (Narcofol, 10 mg/ml, cp-pharma® maintenance: isoflurane (Isofluran cp, cp-pharma®). The dog was ventilated with intermittent positive pressure.

**Figure 2:**
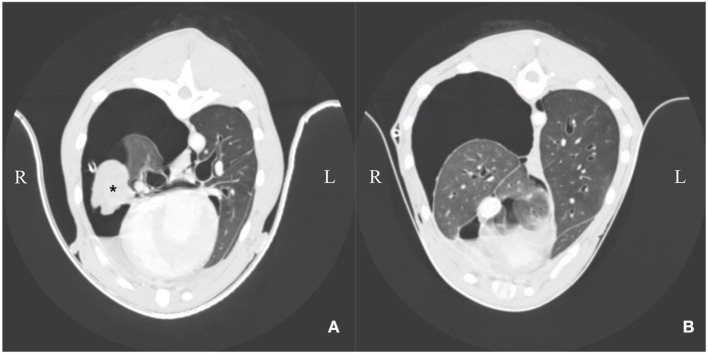
**(A,B)** Day 3: Computer tomography: Heterogenous pleural thickening of multiple lung lobes is seen. The right middle lung lobe is severely atrophic and atelectatic (asterisk). The remaining lung lobes are expanded to ~40% on the right side. Left lung lobes are nearly completely re-expanded on the left side. Mild pleural effusion is present (right > left).

Computer tomographic examination revealed a heterogeneous pleural thickening of multiple lung lobes suggestive of pleural fibrosis. The right middle lung lobe was severely atrophic and atelectatic. The remaining lung lobes were only expanded to 40% on the right side. Left lung lobes were inflated up to 80%. Mild pleural effusion (right > left) was present (Figures 2A,B). Findings were consistent with hydropneumothorax. Contrast-enhanced computer tomography was unremarkable. Lymphangiography of the thoracic duct showed no extravasation of contrast medium.

**Figure 3:**
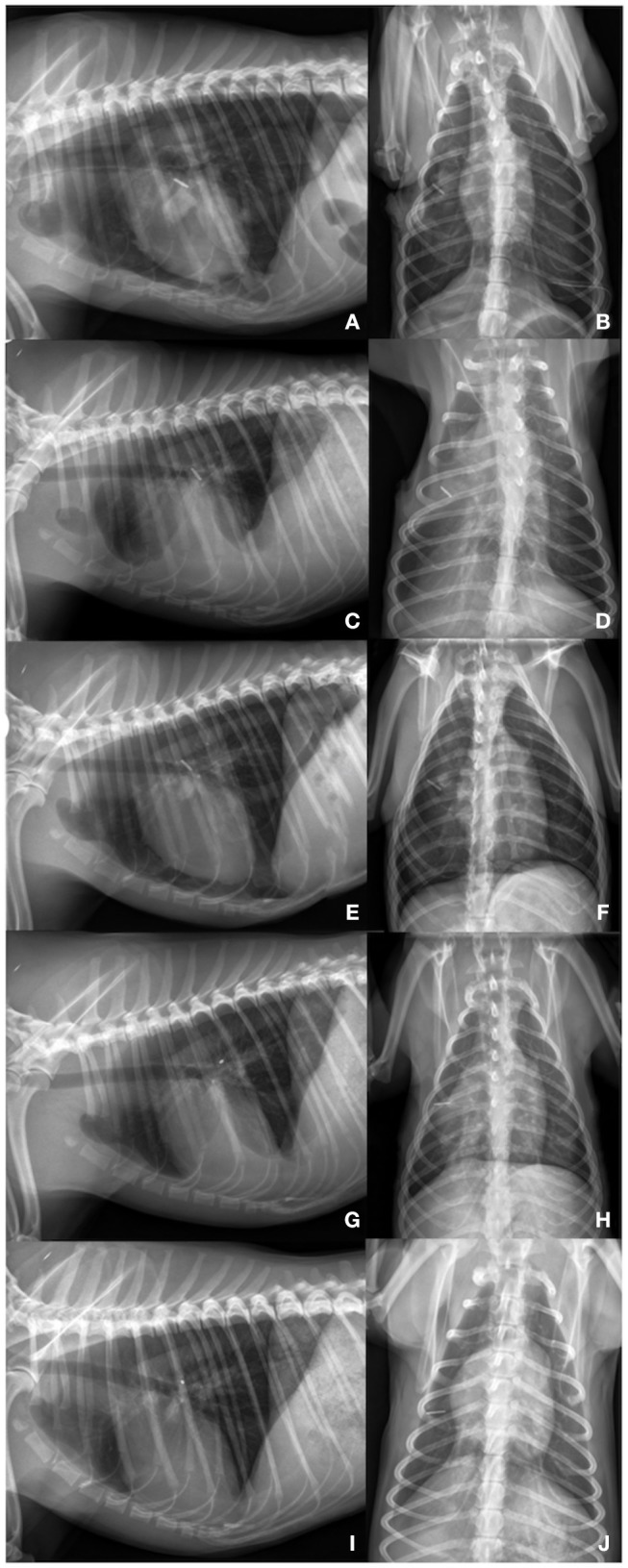
**(A,B)** Day 6: Lateral left sided and ventrodorsal thoracic radiograph view: Lung lobes re-expanded to 80% on the left and right side. Titanum clips are present on the height of the right middle lung lobe. On the left side a chest tube is placed. Pleural thickening is present in all remaining lung lobes. Cardiac silhouette is slightly detached from the sternum. **(C,D)** Day 18: Lateral left sided and ventrodorsal thoracic radiograph view: Moderate increased soft- tissue opacity with partially silhouetted cardiac silhouette. Lung lobes are mildly retracted (90% re-expansion). On the left side a chest tube is present. **(E,F)** Day 24: Lateral left sided and ventrodorsal thoracic radiograph view: After needle thoracocentesis: Lung lobes are expanded to ~80% on the left and 70% on the right side. Heterogenous pleural thickening of the remaining lung lobes is seen. **(G,H)** 4 months later: Lung lobes are re-expanded to 95% on both thoracic sides. Mild pleural fibrosis is present. Mild fluid accumulation is seen. **(I,J)** 15 months later: Lateral left sided and ventrodorsal thoracic radiograph view: Complete re-expansion of all lung lobes. Mild pleural fibrosis is still present. Material of soft-tissue opacity is seen overlaying the cardiac silhouette. Fine needle aspiration revealed the presence of fibrotic tissue. Fluid accumulation could be excluded by ultrasonography.

After imaging, right lateral thoracotomy and a single paracostal approach was performed. To maintain analgesia, fentanyl (Fentadon®, 50 μg/ml, Dechra) was given as a constant rate infusion (0.01 mg/kg/h). Presurgical antibiotics were given (Amox/Clav HEXAL® i.v. 500/100 mg; 12.5 mg/kg IV) and re-dosed after 90 min. Pleural fibrosis presented as withish-tan material overlaying the lung lobes, preventing their re-expansion. Thoracic duct ligation, ablation of the chylic cisterna and a subtotal pericardiectomy were carried out. Due to its highly abnormal appearance, the middle lung lobe was resected. Both thoracic sides communicated well, thus only the left chest tube with a non-return valve (Heimlich type, LEO-ventil, Eickemeyer) was left. Chest tube functionality was assessed clinically, by measurement of produced fluid, active suction q24h and by thoracic radiographs (Figures 3A,B).

Bacterial culture of the resected middle lung lobe was negative. Histology revealed severe, chronic-active, proliferative, and beginning granulomatous pleuritis. Additionally, a moderate to severe subacute to chronic, purulent proliferative, and beginning fibrotic pericarditis with predominantly fibrotic deposits on the serosal lining was diagnosed. A sub-acute to chronic granulomatous, partially demarcated coagulative necrosis of the parenchyma of the middle lung lobe with partially massive and predominantly resorbed hemorrhages, thrombosed vessels, and micro-fragmented bronchi were detected. With periodic acid-Schiff reaction no foreign material, fungal, or parasitic structures were detected (11). Ziehl-Neelsen staining was negative. These findings suggested that the sterile inflammatory process had occurred for at least several weeks, likely much longer. Idiopathic chylothorax with granulomatous pleuritis and highly atelectatic middle lung lobe was diagnosed.

Further patient management included a continuous rate infusion with balanced crystalloids and analgesics (2 ml/kg/h Sterofundin, B. Braun Melsungen AG, 0.002 mg/kg/h fentanyl (Fentadon, 50 μg/ml, Dechra), 0.12 mg/kg/h ketamine (Anesketin, 100 mg/ml, Dechra), and 0.2 mg/kg/h lidocaine (LidoCARD, 2%, B. Braun) for 2 days (12). During inpatient treatment the dog was clinically unremarkable. Starting at day three after thoracotomy meloxicam (Metacam, Boehringer Ingelheim; initially 0.2 mg/kg SC, q24h then 0.1 mg/kg, q24h PO) was administered for 4 months. During this time no gastroprotective agents were given. Amoxicillin clavulanate (12.5 mg/kg q12h PO) was administered for 3 days until bacterial culture confirmed negativity. The dog tolerated meloxicam well and did not show any side effects.

Lungs re-expanded to ~80% 6 days after surgery (Figures 3A,B). Ten days after thoracotomy, the remaining chest tube was removed. The dog was discharged 7 h after chest tube withdrawal with a normal breathing pattern and a SpO2 of 98%. Clinical, radiographic and ultrasonographic follow-up examinations were performed every two to 14 days within the first two months. Further controls were carried out 2, 4, 6, and 15 months later. Pleural effusion persisted for 4 months (Figures 3C–H). Seven days postoperatively effusion specimen was generated from the chest tube. Effusion was diagnosed as non-chylic, non-septic, serosanguineous exudate (cell count: 16,146^*^10^9^/L; protein content: 14 g/L; triglyceride effusion: 0.21 mmol/L; triglyceride serum: 0.70 mmol/L). On day 24, modified transudate (cell count: 3,566^*^10^9^/L; protein content: 28 g/L) and 3.5 months later transudate was present (cell count: 1,698^*^10^9^/L; protein content: 13 g/L). Both effusion specimens were obtained by needle thoracocentesis. Lungs were inflated to ~95% 4 months after initial presentation. Fifteen months post initial presentation, complete re-expansion of the lungs was seen. Pleural effusion was evident neither on radiographs (Figures 3I,J) nor by ultrasonography. Mild pleural fibrosis persisted. Material of soft-tissue opacity overlaid the cardiac silhouette. Fine needle aspiration was performed, and fibrotic tissue was suggested. During the whole observation period the dog was clinically unremarkable. A telephone conversation with the owners 5 years later revealed that the dog is clinically healthy.

## Discussion

Pleural fibrosis develops when pleural effusions irritate the lungs' pleura. Fibrin formation is promoted by the release of chemokines and cytokines (e.g., TGF-β and TNF-α) (13–17). Although some aspects of pathophysiology are already known, it is still unclear why some patients develop pleural fibrosis and others do not (13).

Various causes of pleural fibrosis in dogs are described. One dog suffered from granulomatous pleuritis, which is a form of pleural fibrosis (18). Bacterial culture detected *Pasteurella multocida* within the pericardial effusion. Antibiotic therapy was introduced but 9 months later *Actinomyces bovis* was detected in the thoracic cavity. Due to guarded prognosis the animal was euthanized (2). Another dog with pleural fibrosis was infected with *Serratia* spp. after thoracocentesis. Despite adequate antibiotics, the dog died 2 days after initial presentation (5). Further known causes of pleural fibrosis in dogs are presence of chylothorax (*n* = 3) and traumatic hemothorax (*n* = 1) (3, 5, 7). In cats, chylothorax is the most common cause of pleural fibrosis (*n* = 20; Table 1). One case each was described with feline infectious peritonitis and lymphoblastic lymphoma (FeLV positive) (Table 1). In the present case, pleural alterations hindered the lungs to completely re-expand and were caused by chylic effusion. Neither by bacterial culture nor by histopathological examination infectious agents were detected. Therefore, idiopathic chylothorax was diagnosed (9).

In general, pneumothorax can be classified in dogs as traumatic (e.g., dog bite), spontaneous (e.g., blebs or bullae), or iatrogenic (e.g., after thoracocentesis). Spontaneous pneumothorax is defined as a non-traumatic and closed pneumothorax. It can even occur after laparoscopic ovariectomy (19, 20). All these kinds of pneumothorax are characterized by the presence of supraatmospheric intrapleural pressure (19). In contrast, negative intrapleural pressure is seen in a state called pneumothorax ex vacuo, which is associated in human medicine with an entity called trapped lungs. This entity is one of two different types of unexpandable lungs. This condition occurs in patients with remote pleural inflammation due to infections, hemothorax, pneumothorax, uremia, rheumatoid pleurisy, or thoracic interventions. Negative intrapleural pressure hinders the lungs to re-expand. Effusion is of transudative quality. Patients are mostly asymptomatic. In contrast, lung entrapment is associated with an active pleural inflammation due to active pleural infections or malignant processes. Patients are presented with dyspnea and pleurisy. Intrapleural pressure can be positive and pleural effusions are exudative (10, 21). In human medicine, pleural manometry is recommended to estimate intrathoracic pressure and to distinguish between these two entities (22).

However, a clear distinction is difficult. One study proposed that lung entrapment leads to pleural fibrosis. Subsequently, trapped lung can develop, if the initial insult is resolved (21). It is likely that this occurred in our dog. Actively formed chylothorax led to lung entrapment, which resulted after successful surgery in trapped lung with passively formed transudate and pneumothorax ex vacuo. Manometry was not performed, because it was unavailable.

To what extent middle lung lobe atrophy and necrosis contributed to lung entrapment or trapped lung is questionable. Two scenarios are possible. First scenario is that alterations of the middle lung lobe resulted from lung lobe torsion as a consequence of severe pleural effusion or trauma (23). A second possibility is that middle lung lobe atelectasis resulted from increased pressure on lungs' parenchyma due to severe pleural effusion (24). Both options could not be proven by computer tomography or by pathohistological examination.

Patients suffering from pneumothorax ex vacuo may be mostly asymptomatic. This is consistent with our case and underlines the presence of trapped lung (10). In contrast, previously reported canine and feline cases described that patients suffered mostly from severe respiratory distress (5, 10, 13). This would support the assumption that lung entrapment overweighed in these cases. However, a distinction between trapped lungs and lung entrapment was not done.

The main goal should be to rule out underlying disease processes. Successful treatment hinders further irritations of the pleura and thus progression of fibrin formation (7, 25). As a standard, complete blood cell count, serum biochemistry, effusion analysis including measurements of triglycerides and cholesterol in comparison with serum values, cytology, aerobic and anaerobic culture, thoracic radiographs, and echocardiography should be performed (26). Radiological findings can be suggestive for pleural fibrosis. One study proposed, that two out of three following radiological findings should be present: first pleural thickening, second rounded lung lobes and/or third persistently retracted lung lobes (6). All these alterations were detectable in our dog. Computer tomography is helpful to outline the degree of pleural fibrosis, degree of lung's expansion, course and branching of the thoracic duct, or malignant processes (27). Intra operative presentation is also indicative for pleural fibrosis. Withish-tan material overlays the lungs with variable degrees of adhesion to other intra-thorax structures (5, 6). Despite the presence of fibrous peel surrounding the lungs' parenchyma, it was certainly advantageous for our patient's outcome that no further adhesions were present. To confirm a diagnosis and also to exclude possible underlying diseases, biopsies and pathohistological analysis should be carried out.

In patients with pneumothorax ex vacuo, chest tube placement is not appropriate, because this kind of pneumothorax results from a re-equilibration of intra- and extra-pulmonary pressure (10). Decortication is only recommended in humans and pets with severe respiratory distress, because clinical signs can be fatal. Pneumothorax, tracheal tears, hemorrhages, re-expansion pulmonary edemas, or death can occur (5, 7). Our patient benefited of chest tube placement, because further accumulation of chylic effusion and lung entrapment was reduced. Decortication was not performed, because clinical signs and intraoperative findings were not severe.

To inhibit further inflammation, glucocorticoids were given in cats with variable outcomes (6). In humans, these are only indicated and successful in rheumatoid pleurisy (13). No other inflammatory drugs are described in clinical patients. Experimental studies in mice and rats showed that the NSAID meloxicam had anti-fibrotic properties in kidneys, liver, lung, and pleurisy (28–31). Glucocorticoids were not given in our patient, because wound healing could be delayed, and risk of infection can be increased. Side-effects like polyuria, polydipsia, and panting can also be severe in some dogs. Therefore, meloxicam was given for 4 months until transudative effusion was seen. Pneumothorax ex vacuo resolved, but mild pleural fibrosis persisted. After withdrawal of meloxicam pleural effusion did not reoccurred. It is likely that meloxicam encouraged pleural remodeling and lung expansion, although complete resolution of pleural fibrosis was not achieved. However, NSAIDs should be used cautiously. Gastrointestinal signs like vomitus and diarrhea are the main side effects of the COX-1 sparing meloxicam. The incidence of adverse reactions is inconsistently described (32). Clinical signs were obvious in up to 25% of patients with varying treatment durations (1–84 days) and dosages (33–37). Thus, comparability of data is difficult. However, an increased duration of meloxicam administration is not necessarily associated with a higher incidence of side effects. Administration up to 84 days resulted in adverse effects in 3.4% of dogs, whereas a treatment duration of 28 days in another study caused side effects in 25% of dogs (34, 37). The longest duration of administration was reported in a safety study of transmucosal meloxicam application. Meloxicam was given 26 weeks with up to the fifths time of maximum proposed dosage. Gastrointestinal signs were mainly obvious at higher dosages (38). To ensure drug compatibility in our dog periodic clinical, hematology and blood biochemistry examinations were performed. Additionally, the owners were instructed to closely monitor the dog's behavior, appetite and feces. Currently, prophylactic administration of gastroprotective drugs was not proven to be beneficial, if NSAIDs were given (39). Our dog did not show any obvious side effects, thus, no gastroprotective agents were administered.

Prognosis in dogs and cats with pleural fibrosis is generally poor. In the veterinary literature a high mortality rate is reported. This contrasts with our patient, who had an excellent outcome. It is evident that further investigations are necessary to characterize canine lung trapping and entrapment lungs in detail. The effectiveness of meloxicam in canine granulomatous pleuritis should be ruled out in further clinical studies. However, results of this presented case are promising.

## Concluding Remarks

To the authors' knowledge, this is the first case report with pneumothorax ex vacuo caused by sterile granulomatous pleuritis and chylothorax in a dog with an excellent clinical outcome.

Meloxicam seemed to encourage pleural remodeling and lung expansion, although complete resolution of pleural fibrosis was not achieved.

## Data Availability

All datasets generated for this study are included in the manuscript.

## Ethics Statement

The dog detailed in the case report was presented at the Small Animal Clinic at the Freie Universität Berlin because of severe dyspnea. The owners signed a consent form to permit the diagnostic procedure, treatment on the dog, and to use the collected clinical data.

## Author Contributions

SR contributed to writing the manuscript and review, undertook, additionally, the emergency and postoperatively treatment, and made the control examinations. GM and SR interpreted and described the imaging. GM was the primary surgeon and contributed to writing the manuscript. AG performed the pathohistological examination and contributed to writing the manuscript. BK contributed to dog's treatment and writing the manuscript.

### Conflict of Interest Statement

The authors declare that the research was conducted in the absence of any commercial or financial relationships that could be construed as a potential conflict of interest.

## Abbreviations

DLH: domestic long hair
DSH: domestic short hair
IV: intravenous
N.a.: not approved
PO: per os
RI: reference interval
SC: subcutaneous.
